# Spatial perception mediated by insect antennal mechanosensory system

**DOI:** 10.1242/jeb.243276

**Published:** 2022-02-24

**Authors:** Nwuneke Okereke Ifere, Hisashi Shidara, Nodoka Sato, Hiroto Ogawa

**Affiliations:** 1 Graduate School of Life Science, Hokkaido University, Sapporo 060-0810, Japan; 2 Department of Biological Sciences, Faculty of Science, Hokkaido University, Sapporo 060-0810, Japan

**Keywords:** Cricket, Escape behavior, Multisensory, Air current, Collision avoidance

## Abstract

Animals perceive their surroundings using various modalities of sensory inputs to guide their locomotion. Nocturnal insects such as crickets use mechanosensory inputs mediated by their antennae to orient in darkness. Spatial information is acquired via voluntary antennal contacts with surrounding objects, but it remains unclear whether the insects modulate behaviors mediated by other sensory organs based on that information. Crickets exhibit escape behavior in response to a short air puff, which is detected by the abdominal mechanosensory organs called cerci and is perceived as a ‘predator approach’ signal. We placed objects of different shapes at different locations with which the cricket actively made contact using its antennae. We then examined the effects on wind-elicited escape behavior. The crickets changed their movement trajectory in response to nearby objects such as walls so that they could avoid collision with these obstacles even during the cercal-mediated behavior. For instance, when a wall was placed in front of the crickets so that it was detected by one antenna, the escape trajectory in response to a stimulus from behind was significantly biased toward the side opposite the wall. Even when the antenna on the free side without the wall was ablated, this collision avoidance was also observed, suggesting that the mechanosensory inputs from one antennae detecting an object edge would be sufficient to perceive the location of obstacle in front. This study demonstrated that crickets were able to use the spatial information acquired with their antennal system to modify their behavior mediated by other sensory organs.

## Abstract (precis)

**Summary:** Crickets acquire spatial information such as shape, location and orientation of objects mediated by their antennal mechanosensory system, and even one antenna provides enough information to change their behavior.

## INTRODUCTION

Animals perceive their surroundings using various sensory inputs to guide their locomotion appropriately. In situations in which visual cues are not available, such as in darkness or in very tight spaces owing to surrounding objects, mechanosensory inputs provide effective cues to guide their path. For example, rodents employ their facial whiskers as a tactile sensor array to guide their locomotion (Prescott et al., 2011; Grant et al., 2018). In insects, the mechanosensory cues provided by antennae play an important role in guiding their locomotion. For example, cockroaches walk along a wall by making contact with the wall using their antenna (Camhi and Johnson, 1999; Mongeau et al., 2013). These facts suggest that insects use mechanosensory inputs mediated by their antennae for appropriate course control in various environments (Staudacher et al., 2005).

However, it remains unclear whether insects can use the spatial information of the surroundings perceived with their antennal system to modulate a behavior mediated by other sensory organs. Movement modulation in an oriented behavior adapting to the surrounding space would require the integration of different sensory inputs, one of which induces the behavior itself while the other provides spatial information of the surroundings. To confirm the general use of spatial perception in a mobile behavior, it is necessary to examine whether the spatial context such as the arrangement of objects affects the oriented behavior mediated by other sensory organs, rather than the behavior directly induced by the detected objects. This is because if the animal is reflexively oriented to the object itself that causes the action, it can change its behavior depending on the position of the object without spatial perception. For example, the impacts of antennal stimuli on phonotaxis in female crickets have been investigated. Active contact with an object by the antennae of the crickets performing phonotaxis reduces forward velocity toward the sound source and suppresses phonotactic steering depending on the side where the object is located and the distance (Haberkern and Hedwig, 2016). This finding suggests that the spatial perception of the cricket antennal mechanosensory system may affect the oriented behavior elicited by the stimulus mediated by other sensory organs. To address this issue, we used their escape behavior in response to short airflow, which was detected by the cerci, an abdominal mechanosensory organ (Gras and Hörner, 1992; Tauber and Camhi, 1995; Oe and Ogawa, 2013).

Escape behavior is a distinctly oriented locomotion in which animals move in the opposite direction to threats, such as predators, in order to increase, as much as possible, their distance from the threat (Card and Dickinson, 2008; Domenici et al., 2011a,b). Escape trajectories are often modulated depending on the environmental context (Domenici, 2010; Evans et al., 2019). When goldfish visually perceive an obstacle, they change their escape trajectory to avoid collision with it (Eaton and Emberley, 1991). Escape directionality of lizards and mice also depends on presence and location of shelter, detected visually and auditorily (Hennig, et al., 1976; Vale et al., 2017). The combination of mechanosensory and visual cues results in the escape of rockpool prawn over longer distances and with greater directionality when compared to those triggered by mechanosensory stimulus alone (Guerin and Neil, 2015). Also, in wind-elicited escape behavior, crickets alter their escape trajectory elicited by a short air puff depending on acoustic context represented as different sound frequencies (Fukutomi et al., 2015; Fukutomi and Ogawa, 2017). In addition, a wind stimulus applied to cockroaches that make contact with the wall using their antenna elicits different turning behavior when compared with animals that do not make contact (Ritzmann et al., 1991). Thus, the wind-elicited escape behavior that is mediated solely by the cerci, a different mechanosensory organ from the antennae, would be an ideal model to test the ability of antennal system in spatial perception-based behavioral modulation.

In this study, crickets were tethered on an air-lifted treadmill, and objects of different shapes – a cylindrical rod or a plate – were placed at different distances and in different positions with respect to the antennae. In this condition, the tethered crickets were able to detect the object by actively making contact with the object using their antennae. We compared the escape walking triggered by an airflow stimulus between different conditions with and without antennal stimulation. We found that crickets were able to change their escape trajectory in response to nearby objects such as a wall, suggesting that they could perceive the shape and position of the surrounding obstacles and use this spatial information to modulate the oriented behavior mediated by the other sensory organs. And crickets could detect the edge of objects only with their unilateral antenna to perceive a free space where obstacles were absent on the escape path. These findings suggest a spatial perception ability of the insect antennal system.

## MATERIALS AND METHODS

### Animals

We used the wild-type strain of field crickets for this study (*Gryllus bimaculatus* De Geer 1773, Hokudai WT; Watanabe et al., 2018). The laboratory-bred adult male crickets (0.50–1.00 g body mass) were used in all experiments. They were reared under 12 h:12 h light:dark conditions at a constant temperature of 27°C. The guidelines of the Institutional Animal Care and Use Committee of the National University Corporation, Hokkaido University, Japan, specify no particular requirements for the treatment of insects in experiments. Before commencing the experiments, all crickets were checked to ensure that the legs, cerci and antennae were intact. All experiments were conducted in the early hours of the animals' subjective night at room temperature (26–28°C).

### Treadmill system

**Fig. 1.**
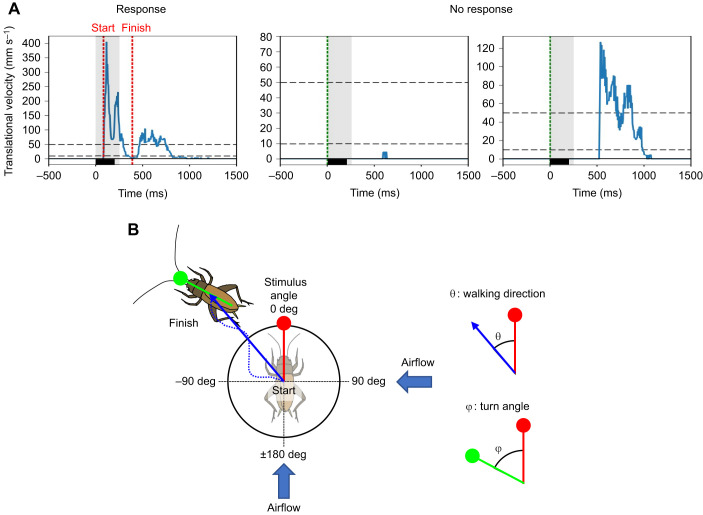
**Definition of behavioral response to the air puff.** (A) Typical time courses of walking velocity (blue traces) in response to the air puff for 200 ms (lower black traces). Based on their time course and magnitude, a trial was determined as ‘response’ or ‘no response’. As shown in the left panel, if a cricket started to walk and its maximum walking velocity was >50 mm s^−1^ (upper black dashed lines) within 250 ms after the stimulus onset (indicated by the gray-shaded area), that trial was classified as a ‘response’. The start of the initial response was defined as the first time when the translational velocity exceeded 10 mm s^−1^ (lower dashed lines) after stimulus onset; the finish of that was defined as the time when the velocity was less than 10 mm s^−1^ after the velocity exceeded 50 mm s^−1^ (red dashed lines). If a cricket did not move (center panel) or began to walk 250 ms or longer after the stimulus onset (left panel), those trials were classified as a ‘no response’. (B) Definition of stimulus angle, walking direction (θ) and turn angle (ϕ) in the initial response to an air-puff stimulus. The left diagram shows the crickets at the start and finish points of the initial response and the walking trajectory on the virtual plane. The walking direction was measured as the angle between the body axis at the start point (red line) and the line connecting the start and finish points of the initial response (blue arrow). The turn angle was measured as the angle made by the body axes at the start and finish points (green line). Both walking direction and turn angles were arranged for forward as 0 deg, clockwise as plus and counterclockwise as minus.

We monitored a cricket's locomotion in response to an air-puff stimulus with the same spherical-treadmill system that was installed within a sound-proofed dark box, as used in our previous studies (Oe and Ogawa, 2013; Fukutomi et al., 2015; Fukutomi and Ogawa, 2017). An animal was tethered on top of an air-lifted Styrofoam ball (diameter=60 mm) using a pair of L-shaped insect pins that were stuck to the cricket's tergite with paraffin wax. The cricket's walking activity was monitored as rotation of the ball at a sampling rate of 200 Hz, using two optical mice that were mounted orthogonally around the ball. TrackTaro software (Chinou Jouhou Shisutemu Inc., Kyoto, Japan) was used to measure the movement trajectory and to calculate parameters such as translational and angular turn velocities based on the measured ball rotation (Fig. 1).

### Air-puff and tactile stimulations

Crickets detect a surrounding airflow by using the cerci that are abdominal mechanosensory organs and exhibit an oriented escape behavior in response to short air puff (Gras and Hörner, 1992; Tauber and Camhi, 1995; Oe and Ogawa, 2013). To induce the escape behavior, an airflow stimulus was provided to the cerci of the cricket that was stationary for more than 1 s by a short puff of nitrogen (N_2_) gas from a plastic nozzle (15 mm diameter) connected to a PV820 pneumatic picopump (World Precision Instruments, Sarasota, FL, USA). By adjusting the delivery pressure of the picopump, the velocity of the air puffs was controlled at 0.68 m s^−1^, which was measured at the center of the treadmill with a 405-V1 thermal anemometer (Testo, Yokohama, Japan). The duration of the air-puff stimulus was set to 200 ms. Eight air-puff nozzles were arranged around the inside wall of the arena, and the height of the nozzles was aligned with the same horizontal plane as the animal. The nozzle ends were positioned at a distance of 130 mm from the center of the treadmill and were spaced 45 deg apart. In the experiments to test the effects of antennal tactile stimulation, the air-puff stimulus was applied from either the posterior (180 deg) or lateral side (90 deg) of the animal. In a preliminary experiment to test the effects of bilateral ablation of antennae on the escape behavior, the stimulus was provided in sequence from eight nozzles that were spaced 45 deg apart (Fig. S1).

**Fig. 2.**
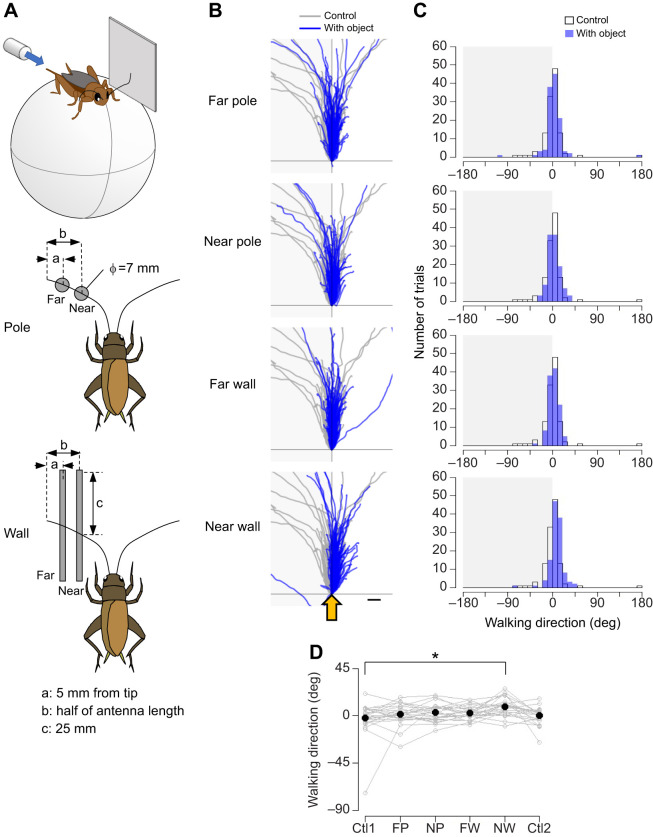
**Effects of antennal mechanosensory inputs on the escape response to airflow from behind.** (A) Experimental design. A cylindrical pole (diameter=7 mm) or square plate (50×50 mm) was positioned at the anterolateral position at different distances from the cricket. Far: 5 mm from the tip of the antenna. Near: half of the antenna length. A puff of air was applied from behind the cricket. (B) Walking trajectories in the initial response to the air puff combined with the antennal stimulation. Gray traces show the trajectories under control condition with no objects. Blue traces show the trajectories under antennal stimulation conditions. Shaded region indicates the side on which the objects were placed. Yellow arrows indicate the direction of the air puff. Scale bar indicates 10 mm. (C) Distributions of walking direction under different conditions. Open and blue bars indicate the data from the control and antennal-stimulation conditions, respectively. (D) Walking direction under different conditions. *N*=24 individuals. Five trials were implemented for each condition for each individual. Gray open circles connected by lines indicate mean values for all trials in each individual. Black dots denote the average across the population for each condition. Data for the control condition were obtained for each individual twice, at the beginning (Ctl1) and end (Ctl2) of the experiment. FP, far pole; NP, near pole; FW, far wall; NW, near wall. *P<0.05 (Fisher's nonparametric test with Bonferroni correction).

**Fig. 3.**
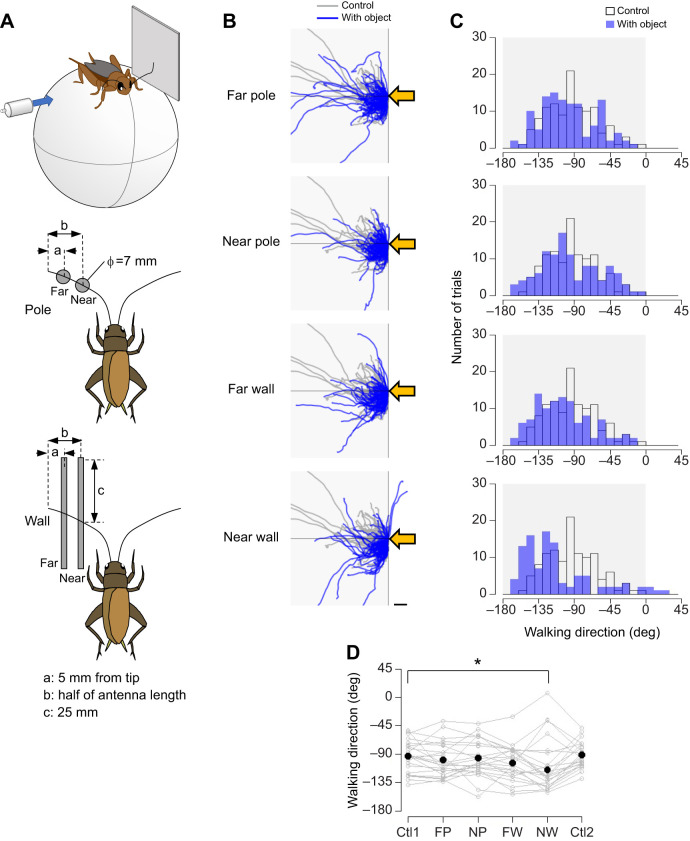
**Effects of antennal mechanosensory inputs on the escape response to airflow from the side contralateral to obstacles.** (A) Experimental design. As in Fig. 2, a cylindrical pole (diameter=7 mm) or square plate (50×50 mm) was positioned at the anterolateral position at different distances from the cricket. Far: 5 mm from the tip of the antenna. Near: half of the antenna length. Air puff was applied from the side contralateral to the objects. (B) Walking trajectories in the initial response to the air puff combined with the antennal stimulation. Gray and blue traces show the trajectories under control and antennal-stimulation conditions, respectively. Shaded region indicates the side on which the objects were placed. Yellow arrows indicate the direction of the air puff. Scale bar indicates 10 mm. (C) Distributions of walking direction under different conditions. Open and blue bars indicate the data from the control and antennal-stimulation conditions, respectively. (D) Walking direction under different conditions. *N*=24 individuals. Five trials were implemented for each condition for each individual. Gray open circles connected by lines indicate mean values for all trials in each individual. Black dots denote the average across the population for each condition. Data for the control condition were obtained for each individual twice, at the beginning (Ctl1) and end (Ctl2) of the experiment. FP, far pole; NP, near pole; FW, far wall; NW, near wall. *P<0.05 (Fisher's nonparametric test with Bonferroni correction).

**Fig. 4.**
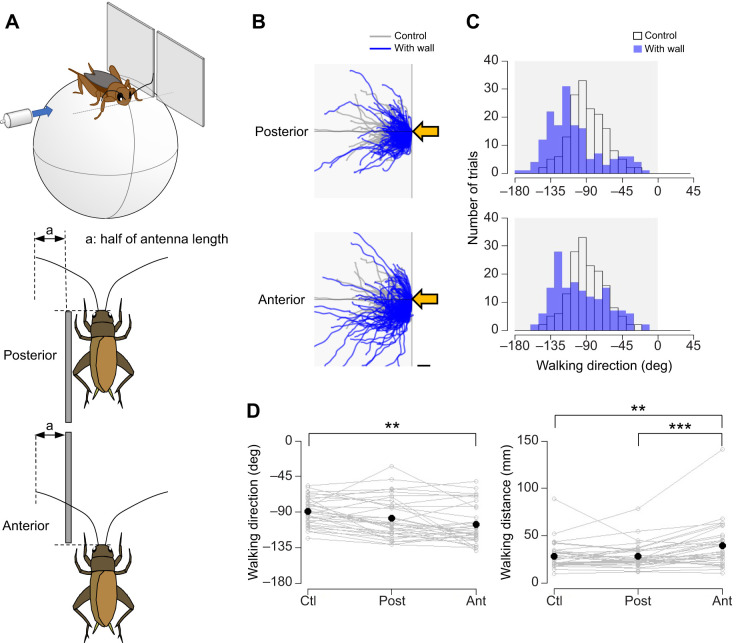
**Positional effects of the side wall on the wind-elicited escape behavior.** (A) Experimental design. A square plate (50×50 mm) was placed laterally at the anterior or posterior position of the cricket. A puff of air was applied from the side contralateral to the plate. (B) Walking trajectories in the initial response to the air puff combined with the antennal stimulation. Gray and blue traces show the trajectories under the control and antennal-stimulation conditions, respectively. Shaded region indicates the side on which the plate was placed. Yellow arrows indicate the direction of the air puff. Scale bar indicates 10 mm. (C) Distributions of the walking direction under different conditions. Open and blue bars indicate data from the control and stimulation conditions, respectively. (D) Walking direction and distance under different conditions. *N*=33 individuals. Five trials were implemented for each condition for each individual. Gray open circles connected by lines indicate mean values for all trials in each individual. Black dots denote the average across the population for each condition. Ctl, control; Post, posterior; Ant, anterior. **P<0.01, ***P<0.001 (Fisher's nonparametric test or Wilcoxon signed-rank test with Bonferroni correction).

**Fig. 5.**
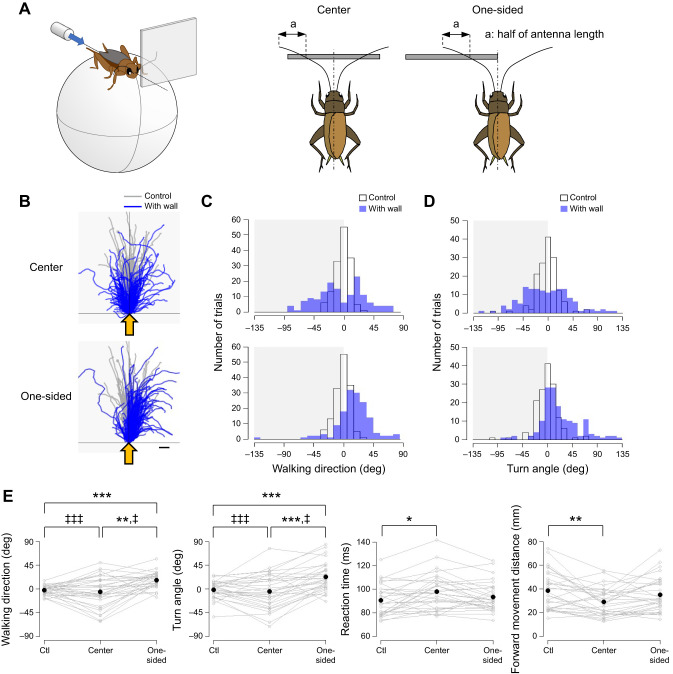
**Positional effects of the wall placed in front of the cricket on the wind-elicited escape behavior.** (A) Experimental design. A square plate (50×50 mm) was positioned in front of the cricket either at the center or toward one side. A puff of air was applied from behind the animal. (B) Walking trajectories in the initial response to the air puff combined with the antennal stimulation. Gray and blue traces show the trajectories under the control and antennal-stimulation conditions, respectively. Shaded region indicates the side on which the plate was placed. Yellow arrows indicate the direction of the air puff. Scale bar indicates 10 mm. (C,D) Distributions of walking direction (C) and turn angle (D) under different conditions. Open and blue bars indicate data from the control and stimulation conditions, respectively. (E) Walking direction, turn angle, reaction time and forward movement distance under different conditions. *N*=30 individuals. Five trials were implemented for each condition for each individual. Gray open circles connected by lines indicate mean values for all trials in each individual. Black dots denote the average across the population for each condition. Ctl, control. *P<0.05, **P<0.01, ***P<0.001 (Fisher's nonparametric test and Wilcoxon signed-rank test with Bonferroni correction). ^‡^*P*<0.05, ^‡‡‡^*P*<0.001 (Wallraff test with Bonferroni correction).

**Fig. 6.**
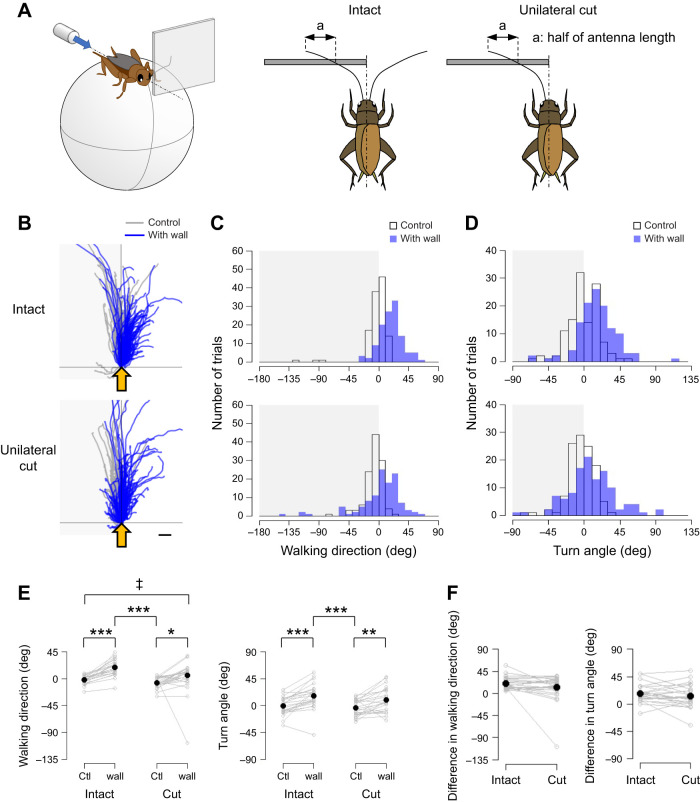
**Unilateral lesion of the antenna had little impacts on the modulation of escape trajectory when an obstacle was placed in front of the animal.** (A) Experimental design. A square plate (50×50 mm) was positioned on one side in front of the cricket. A puff of air was applied from behind the animal. In unilateral-cut condition, the one antenna contralateral to the plate was resected. (B) Walking trajectories in the initial response to the air puff combined with the antennal stimulation in intact (upper) and antenna-ablated crickets (lower). Gray and blue traces show the trajectories under the control and antennal-stimulation conditions, respectively. Shaded region indicates the side on which the plate was unilaterally placed. Yellow arrows indicate the direction of the air puff. Scale bar indicates 10 mm. (C,D) Distributions of the walking direction (C) and turn angle (D) under different conditions in intact (upper) and antenna-ablated crickets (lower). Open and blue bars indicate data from the control and antennal stimulation conditions, respectively. (E) Walking direction, turn angle, reaction time and forward movement distance under different conditions. (F) Angular differences in walking direction (left) or turn angle (right) between control and antennal-stimulation conditions. *N*=24 individuals. Five trials were implemented for each condition for each individual. Gray open circles connected by lines indicate mean values for all trials in each individual. Black dots denote the average across the population for each condition. Ctl, control. *P<0.05, **P<0.01, ***P<0.001 (Fisher's nonparametric test and Wilcoxon signed-rank test with Bonferroni correction). ^‡^*P*<0.05 (Wallraff test with Bonferroni correction).

A vertical cylindrical rod (diameter=7 mm) or square plate (50×50 mm) was placed in different orientations and distances from the antennae. The objects were placed either in the ‘far’ position, which was 5 mm proximal from the tip of the antenna, or in the ‘near’ position, which was at half the antenna length (Figs 2A and 3A). For different orientations of the object, the plate was placed either on the left side or in front of the cricket in the near position. The lateral plate was placed either at the anterior or posterior position (Fig. 4A). The frontal plate was centered or placed on one side in front of the animal (Figs 5A and 6A).

### Video recording of antennal movement

To check the frequency and duration of contact of the antenna with the object, the antennal movement was monitored using a high-speed digital camera (CHU30-B, Shodensha, Osaka, Japan) under red LED illumination. The voluntary movement of the antenna was recorded for 60 s in each trial at a frame rate of 90 frames s^−1^ with a resolution of 640×480 pixels. We manually counted the frames in which the antenna was in contact with the plate and calculated the frequency and duration of contact. We compared the antennal contacts with the anterior and posterior plates positioned on the lateral side of the animal, and also the contacts of ipsilateral and contralateral antennae to the plate positioned on one side in front of the animal. For each condition, five trials of the measurement for 1 min were performed with an interval of 1 min between trials. Ten individuals were recorded in total for each experiment.

### Experimental procedure

The common procedure to record escape movement of crickets using the treadmill system is as follows. At first, a cricket was positioned on top of a Styrofoam ball by using a micromanipulator that moved a pair of L-shaped insect pins attached to its tergite. Next, an object was moved to the specific location against the animal using a manipulator, and then we started recording the movement before and after the air-puff stimulus, including a waiting period to confirm that animal was standing still for 1 s. (The waiting period was 11.57±0.99 s in conditions with object presentation in Figs 2 and 3.) Because the object remained presented at a specific location during experiments in each condition, the animals were able to sense the object by contacting it with their antennae voluntarily during all trials including inter-trial intervals. We adopted three experimental arrangements with different representation of the objects and stimulations.

#### Bilateral antennal cut

To examine the contribution of the antennal inputs induced directly by airflow stimulus to the wind-elicited behavior, we recorded the movement of crickets with intact antennae and those in which the antennae were bilaterally ablated at the base (Fig. S1). Nothing was placed around the crickets and thus tactile stimulation of the antennae was eliminated. In this experiment, a single air puff was delivered from each nozzle positioned at 0, 135, −90, 45, 180, −45, 90 and −135 deg, in that order, with an inter-trial interval greater than 1 min. For each individual, 40 trials each (five trials for each stimulus angle) were recorded before and after the ablation of the antennae. To ensure that the animal had recovered from the damage of the antennal ablation, the experiments using the antenna-ablated crickets were performed more than 40 min after the ablation. Twenty-six individuals were tested in total.

#### Different shapes and distances of object

To test the effects of object shape and distance, we adopted the following five types of stimulation conditions (Figs 2 and 3). The rod was placed near or far from the antenna, referred to as ‘near pole’ and ‘far pole’. The plate was placed near or far from the antenna on the side of the cricket, referred to as ‘near wall’ and ‘far wall’. In the control, neither rod nor plate was placed (control). The air puff was applied from the rear of the cricket or from its lateral side opposite to the objects. For each tactile stimulation condition, 10 trials were performed with an inter-trial interval greater than 1 min. The stimulation conditions were tested in the following order: control, far pole, near pole, far wall and near wall. Finally, the control condition was tested again to confirm that the escape movement was not adapted through the series of the experiment. The trials in this condition were referred to as ‘control2’. In total, 60 trials were performed for each individual. Twenty-four individuals were tested.

To check for a potential turbulence effect of the air-puff stimulation when the plate was positioned on the lateral side of the cricket, we measured the escape behaviors of the cricket in which both antennae were ablated from the base (Fig. S2A–C). After more than 40 min of antennal ablation, the escape responses to the air puff from behind (180 deg) or on the lateral side opposite to the plate (90 deg) were recorded in two different conditions: without the wall (control) and with the wall placed at the near position. For each individual, 20 trials (10 trials for each stimulus angle) were recorded. Twelve individuals were tested in total.

#### Different locations of wall

To test the effects of the object location, we adopted two types of experiments (Figs 4 and 5). In the first type, the plate was placed at the lateral side of the cricket either anteriorly or posteriorly relative to its head, referred as ‘anterior’ and ‘posterior’, respectively, and the air puff was applied from the contralateral side of the plate (Fig. 4). The side edge of the plate was aligned with the base of the antenna. Three stimulation conditions, control, anterior and posterior, were tested in random order for each individual. In the second type of the experiment, the plate was either centered in front of the cricket or placed toward one side, referred as ‘center’ and ‘one-sided’, respectively, and the air puff was applied from its rear (Fig. 5). In the center stimulation condition, the center of the plate was aligned to the midline of the animal so that the cricket could access the plate with both antennae. In the one-sided condition, the side edge of the plate was aligned to the midline of the animal so that the cricket accessed the plate only with the antenna ipsilateral to the plate. The three stimulation conditions, control, center and one-sided, were tested in random order for each individual. In both types of experiments, five trials were performed for each stimulation protocol with an inter-trial interval greater than 1 min. In total, 15 trials were performed for each individual for each type of experiment. Thirty-three and 30 individuals were used in the first and second types of experiments, respectively. For the one-sided stimulation condition in the second type of experiment, the plate was placed on the right side of the animals in 15 crickets, and in another 15 crickets on the left side to counterbalance the stimulation to the antenna bilaterally.

To examine the contribution of bilateral antennae to the behavioral modulation, we adopted the one-sided stimulation condition mentioned above to the unilaterally antenna-ablated crickets (Fig. 6). An air-puff stimulus was applied from the rear of the cricket. The movement of the intact crickets was monitored in the ‘control’ condition without the plate and in the ‘wall’ condition where the plate was placed on one side in front of the cricket. Then, the antenna contralateral to the plate was ablated at the base. After the ablation, the animal was left undisturbed for more than 40 min, after which its movements were recorded under the control and wall conditions. For each condition, five trials were performed with an inter-trial interval greater than 1 min. In total, 20 trials were performed for each individual. Twenty-four individuals were used.

### Data analysis

Behavioral data provided by the TrackTaro software were processed and analyzed offline, using algorithms customized using Python 3.7.1 (Jupyter Notebook version 5.7.4). We first classified all recorded data from all trials into ‘wind-elicited response’ or ‘no response’ based on the walking speed, as used in previous studies using the treadmill system (Oe and Ogawa, 2013; Fukutomi et al., 2015; Fukutomi and Ogawa, 2017). If the walking speed exceeded 10 mm s^−1^ during the period from the stimulus onset to 250 ms after the stimulus onset and the maximum translational velocity was greater than 50 mm s^−1^, the cricket was considered to respond to the air current (Fig. 1A). If the cricket did not begin to move within this response definition period of 250 ms after the stimulus onset, that trial was considered as ‘no response’. The response probability was defined as follows:
(1)

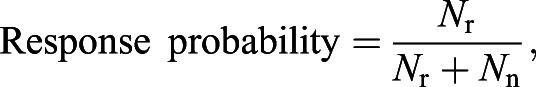
where *N*_r_ and *N*_n_ are the number of trials categorized as a wind-elicited response and a no response obtained for each stimulation condition, respectively. We focused on the initial responses to the air-puff stimulation, of which the response start was defined as the first time when the translational velocity exceeded 10 mm s^−1^ after stimulus onset; the finish was defined as the time when the velocity was less than 10 mm s^−1^ after the velocity exceeded 50 mm s^−1^ (Fig. 1A) (Oe and Ogawa, 2013; Sato et al., 2017, 2019). The periods from the start to finish of the initial response to air puff from behind and side were 435.79±142.1 and 371.60±71.97 ms in the control (no object) condition, respectively (*N*=24 individuals, 5 trials for each individual). We measured four locomotion parameters for this initial response: walking direction, turn angle, reaction time and walking distance. Definition and calculation of these parameters are the same as in our previous studies (Oe and Ogawa, 2013; Fukutomi et al., 2015; Fukutomi and Ogawa, 2017). The walking direction was measured as the angle between the body axis at the start point (red line in Fig. 1B) and the line connecting the start and finish points of the initial response (blue arrow in Fig. 1B). The turn angle was measured as the angle made by the body axes at the start and finish points (green line in Fig. 1B). The walking direction and turn angle were arranged for the forward direction as 0 deg, clockwise as plus and counterclockwise as minus. We arranged the walking direction so that it ranged from 0 to ±180 deg (Fig. 1B). To compare the magnitude of the turning movement, unlike the walking direction, the turn angle was not arranged. The trajectory length of the initial response is referred to as the walking distance. The forward distance was defined as the travel distance of the forward movement during the initial response. The reaction time was defined as the time delay from the opening of the delivery valve in the picopump to the start of the response. Thus, it included a constant travel time of the airflow between the nozzle and the cricket.

### Statistical methods

R programming software (version 3.5.2, R Development Core Team) was used for the statistical analysis. To avoid pseudo-replication, we used the mean value of the data obtained in the trials categorized as wind-elicited response for each individual as the representative value for the statistical tests. Because the walking direction is a circular parameter, we calculated the circular mean angle of the walking direction for each individual. The turn angle was treated as a non-circular parameter just like reaction time and walking distance because it was measured as angular magnitude of rotation movement. All statistical tests for the significant effects among three or more groups were corrected with Bonferroni correction.

Prior to statistical testing of the non-circular parameters including turn angle, walking distance and reaction time, we checked the distribution of the datasets using the Shapiro–Wilk test. As the data for these parameters in all the experiments were not normally distributed, we used the Wilcoxon rank-sum test or Wilcoxon signed-rank test to assess the significance of the stimulation conditions. If some of the tested individuals did not respond, the Wilcoxon rank-sum test to test the unpaired dataset was used. If all tested individuals responded, the Wilcoxon signed-rank test to test the paired dataset was used. The Wilcoxon signed-rank test was also used to assess the significance of the stimulation condition for the response probability.

Fisher's nonparametric test for the common median direction (Fisher, 1993; Pewsey et al., 2013) was used to assess the significance of the stimulation condition for the walking direction. In order to assess the significance of the stimulation condition for the angular dispersion around the mean of the walking direction and turn angle, we used the Wallraff test, for which the package ‘circular’ was used (https://r-forge.r-project.org/projects/circular/).

## RESULTS

### Effects of antennal mechanosensory inputs on the cercal-mediated escape behavior

A short air puff elicits walking or jumping in crickets, which is considered an escape behavior because they move in the direction opposite to the stimulus (Oe and Ogawa, 2013; Fukutomi et al., 2015; Sato et al., 2019). The cercal sensory system, which is an abdominal mechanosensory organ, mediates wind-elicited escape behavior. First, to test whether the cricket's antennae also could sense the airflow stimulus and contribute to the escape behavior, we compared the response to the air puff of intact and bilateral antennae-removed crickets. If the antennal mechanosensory inputs were involved in the wind-elicited escape behavior, ablation of the antenna would alter the response to the stimulus from the anterior because the antenna was more sensitive to the frontal stimulus. Here, the air-puff stimulus was applied from eight angles around the cricket, and the stimulus-angle-related directionality in the escape behavior was examined. The antenna-ablated crickets responded to the air-puff stimuli applied from any angle, and their trajectory did not differ from that of intact crickets (Fig. S1A). The antenna ablation had little effect on either the walking direction or the turn angle for any stimulus angle (Fig. S1B–D). There was also no significant difference in angular dispersion around the mean angles of the walking direction between the cut and intact conditions (Wallraff test; Table S1A). The reaction time was also not affected by antennal ablation (Fig. S1D). The turn angle, walking distance and response probability were not affected except for the specific stimulus angles of 180, 135 and 90 deg, respectively (Fig. S1D, Table S1A). This suggests that antennal ablation had little impact on responsiveness and directional control. In conclusion, our results indicate that any interaction of airflow with antennae did not contribute to the wind-elicited escape behavior of crickets.

Next, we tested whether the crickets could alter the escape behavior when they had the opportunity to place their antennae in contact with potential obstacles. We also examined how the crickets modulated the escape trajectories depending on the object shape and distance. Fig. 2 shows the responses to the air puff from behind when a cylindrical pole or square plate was positioned at different distances (Fig. 2A). The walking trajectories showed that the crickets tended to walk toward the free side, opposite to the objects, and this movement was most pronounced in the near wall condition (Fig. 2B). The data on the walking direction revealed that it was significantly biased toward the contralateral side of the plate in the near wall condition (Fig. 2C,D, Table S1B). This biased effect by the plate placed at the near position was not observed for the spontaneous walking movements before the airflow stimulation (Fig. S2D–F). In contrast, there was no significant effect of either the plate placed at far position or the poles placed at near or far position. The turn angle, reaction time, walking distance and response probability were not affected by the presence of objects regardless of their location (Fig. S3A,B, Table S1B).

We further tested the effects of the pole or plate placed at different distances on the response to airflow applied from the side of the cricket (Fig. 3A). In the control condition with no object, the cricket moved in the direction opposite to the air-puff stimulus, as shown by gray traces in Fig. 3B, and the walking trajectories were distributed around −90 deg (Fig. 3C). However, when the objects were placed on the contralateral side of the stimulus, the cricket moved more backward and the distributions of the walking direction were shifted (Fig. 3C). The backward bias in the walking direction was significant when the plate was positioned at the near position (Fig. 3D, Table S1C). The plate located at the far position also tended to bias the walking direction backwards, but this effect was not significant. The pole did not affect the walking direction, regardless of the location. These results suggest that crickets alter their walking direction to avoid hitting the obstacle depending on the obstacle shape and distance to the obstacles. The modulation in the walking direction would solely result from antennal contacts and not from air turbulence by the objects because the effect of the plate that was located at the near position was abolished by bilateral ablation of the antennae so that the crickets could not detect it (Fig. S2, Table S1D). In contrast, neither the pole nor the plate had any effect on the turn angle, walking distance or response probability regardless of their location (Fig. S3A,B, Table S1C). There was a significant difference in the reaction time between control conditions 1 and 2, which were performed without objects before and after the experiments using other conditions with objects (Fig. S3A,B). This might be due to after-effects of many previous encounters with obstacles. In summary, the results shown in Figs 2 and 3 indicate that the crickets were able to sense the object shape and the distance to it and to modulate escape trajectory to avoid collision with obstacles by using their antennal mechanosensory system.

### Crickets could sense the location of obstacles with their antenna

Next, to test the ability of the antennal system to sense the location of the obstacles, we examined the effects of the location of the plate on the escape behavior. We placed the plate on the side of the cricket at two different positions, anterior or posterior (Fig. 4A). We predicted that crickets would change their movement direction depending on the plate location: they might move backwards when the plate is placed at the anterior position and forward when it is placed at the posterior position. The stimulus was applied from the contralateral side to the plate.

The walking direction was significantly biased backwards by the anterior plate. In contrast to our prediction, the posterior plate had no significant effect and did not enhance forward movement (Fig. 4B–D, Table S1E). But the posterior plate also resulted in bimodal distribution of all trial data in the walking direction and separated the within-individual means into two clusters (Fig. 4C,D), suggesting the possibility that some individuals changed their trajectory by sensing the posterior plate. The bias in the walking direction caused by the anterior plate was coupled with a longer walking distance (Fig. 4D). It is likely that crickets might have to move longer distances in order to avoid the anterior wall. The turn angle, reaction time and response probability were not affected by the presence of the plate in either position (Fig. S3C,D, Table S1E). The lack of an effect of the plate positioned posteriorly might be due to the cricket's failure to detect it. To confirm this possibility, we observed the voluntary movement of the antenna against the plate placed at anterior or posterior position by using a high-speed camera. Although the crickets made fewer contacts with posterior plate than with anterior plate, they still did so on average approximately 200 times during the recording period of 5 min (Fig. S4A). There were no significant differences in the duration of each contact between the anterior and posterior positions of the plate (Fig. S4A, Table S1F). This indicated that the cricket could sense the plate placed at the anterior position more precisely, but it would have been possible to also perceive the posterior plate. The changes in their escape behavior depending on the wall position suggest that crickets can sense the location of the objects with one antenna.

### Bilateral antennal inputs were not always necessary to sense the position of frontal obstacle

The results thus far revealed that crickets could change their escape behavior by locating objects even with only one antenna. This was because it was difficult for the crickets to touch the laterally placed plate and pole with their contralateral antenna. If both antennae were used to detect the object, the crickets could perhaps perceive the object location more precisely. To examine this possibility, we studied the trajectory of the escape response to the airflow from the rear when the plate was placed in front of the cricket at different positions, namely, at the center or to one side (Fig. 5A). In this experiment, the crickets were able to touch the plate at the center in front of them with both antennae, while the plate located to one side could be touched by only the ipsilateral antenna. We predicted that the crickets in response to the airflow from the rear would move forward, biased to the free side and contralateral to the plate, when the plate was placed at one side. As expected, the plate placed on one side significantly biased the walking direction and turn angle to the free side opposite to the wall (Fig. 5B–E, Table S1G).

In contrast, when the wall was placed in front of the animal and aligned to the center, the trajectory was greatly altered. Both the walking direction and the turn angle became widely and bimodally distributed, and the number of movements decreased around 0 deg of the walking direction and turn angle, although there was no significant difference in the median of these directional parameters compared with the control condition (Fig. 5C,D). As a result, the center wall increased the angular dispersion of the walking direction and turn angle significantly compared with the control and one-sided wall conditions (Fig. 5E, Table S1G). Furthermore, there was no significant difference in the walking distance between the conditions (Fig. S5A), but the forward movement was significantly reduced in the center wall condition (Fig. 5E, Table S1G). These results indicate that the center wall might enhance the lateral movement rather than the forward movement. The cricket could alter its escape movement depending on the position of the obstacle to effectively avoid collision with it. In addition, the reaction time significantly increased when the plate was placed centrally in front of the animal (Fig. 5E). It might take a longer time for the cricket to make a decision on the escape direction. The plate placed in the frontal region of the cricket had no effect on the response probability regardless of its position (Fig. S5A, Table S1G), suggesting that the obstacles on the escape route might not suppress the escape decision.

It was revealed that crickets could modulate their escape behavior depending on the position of the obstacle. This then leads us to the question: did the crickets need to compare the left and right antennal inputs to sense the precise plate position? To answer this, we ablated one antenna and examined the modulation of the escape behavior triggered by the stimulus applied from behind when the plate was positioned in the front, ipsilateral to the intact antenna (Fig. 6A). Even though one antenna contralateral to the plate was ablated, the walking direction and turn angle were biased to the object-free side as in intact crickets (Fig. 6B–E, Table S1H). This result indicates that the crickets were able to perceive the position of front wall with only one antenna. It was likely that bilateral antennal inputs were not always required to sense the position of the frontal obstacle. To observe how the intact crickets contacted the one-sided plate with both antennae, we monitored the antennae movements using a high-speed camera. We found that crickets rarely touched the plate with the contralateral antenna (Fig. S4B, Table S1F). Instead, the antenna ipsilateral to the plate actively contacted not only the surface but also its edge. Probably, the crickets, of which one antenna was ablated, detected the medial edge of the one-sided front wall with the other antenna and turned around to avoid colliding with it. In addition, the angular differences in the walking direction and the turn angle between the conditions with and without the plate were calculated to evaluate the bias effects of the one-sided front wall (Fig. 6F, Table S1H). There were no significant difference in those values between before and after the antenna ablation, meaning that the escape movement was modulated as much by mechanosensory inputs from one antenna as it was from bilateral ones. Even in the crickets with one antenna ablated, there was no significant difference in the reaction time, walking distance or response probability with and without the front wall (Fig. S5B, Table S1H). In addition, there was also no significant difference in these parameters between intact and unilaterally antenna-ablated crickets (Table S1H). These results imply that it is not always necessary for crickets to compare mechanosensory inputs from left and right antennae for the object localization.

## DISCUSSION

### Object localization by antennal mechanosensing

In this study, the crickets were tethered on the treadmill so that the experimenter could manipulate the shape, distance and position of the stimulating objects. The crickets modulated their trajectory depending on the distance to the object, the object shape such as plate or rod, and the relationship between the object orientation and stimulus direction. The plate at the near position altered the walking direction, but that at the far position did not. In contrast, the pole had no effect regardless of the distance from the animal (Figs 2 and 3). In addition, the effects of the laterally placed plate differed depending on its anterior–posterior location (Fig. 4), and the plate placed in front of the cricket had different effects on the escape response from that placed laterally (Fig. 5). These results indicated that the crickets were able to perceive not only the objects that they came in contact with, but also their spatial information, including distance, location and orientation.

Cockroaches use the antennal system to guide their locomotion in an environment with obstacles such as barriers and walls (Camhi and Johnson, 1999; Harley et al., 2009; Baba et al., 2010). When cockroaches actively touch an object placed close to their antenna, they exhibit different turning behaviors depending on the horizontal position of the object (Okada and Toh, 2000, 2006). These facts suggest that the cockroaches are able to identify the location and orientation of objects using their antennal mechanosensory system. However, if the antennal inputs directly cause a movement in a specific relationship to the detected object position, the cockroach can avoid or localize an obstacle. Thus, it is difficult to distinguish whether this orienting behavior is caused by the perception of the entire surrounding space or simply by a reflexive response to the tactile stimulation. In contrast, our present study directly demonstrates the spatial perception ability of the crickets' antennal system by examining the escape behavior elicited by airflow stimulus, which was not mediated by the antennae (Fig. S1). Crickets altered their escape behavior even for identical airflow stimulus applied in the same direction, depending on the location and orientation of the object. This suggests that the crickets may be able to perceive the entire arrangement of objects in the surrounding space.

The cricket, which was tethered on the treadmill, actively sensed the objects by moving its antennae freely and scanning the surrounding space. This has also been reported in cockroaches (Okada and Toh, 2006). For spatial perception, ‘active sensing’ is one of the most reliable ways to acquire contextual cues from the environment. In mechanosensory active sensing, animals voluntarily move their sensory organs across the surrounding objects to acquire information about their environment and to enhance the searching space and sampling frequency. Rodents use their facial whiskers not only as passive sensory organs, but also to perceive surrounding objects by actively moving the whiskers (Mitchinson et al., 2007; Deutsch et al., 2012; Voigts et al., 2015; Bush et al., 2016). Insects move their antennae to actively sample the surrounding space and are able to identify obstacles, recognize conspecifics and predators, actively track objects, and probe surface textures (Staudacher et al., 2005; Okada and Toh, 2006). Our results suggest that active sensing by a cricket's antennal system provides advanced spatial information to perceive the surrounding space, which allows the crickets to modulate their behavior mediated by different sensory organs.

### Context dependent modulation of escape behavior

The presence of an object detectable by the antennae altered the direction in which the cricket moved in response to the airflow. This wind-elicited movement is considered to be one of the escape behaviors in which the cricket perceives the airflow as a cue for the approach of a predator (Tauber and Camhi, 1995; Casas and Dangles, 2010; Dupuy et al., 2011). The direction of the wind-elicited escape in crickets is precisely controlled depending on the stimulus angle, similar to the rapid escape in flies and cockroaches (Domenici et al., 2008; Card, 2012). In the absence of an object, as in the control condition, the crickets fled in the opposite direction from which the air-puff stimulus came (Fig. S1; Oe and Ogawa, 2013). Consistent with previous studies, the directions of movement in the initial response to the airflow from either the rear or lateral side that was used in this study were also distributed around the front or contralateral direction to the stimulus (Fukutomi et al., 2015; Sato et al., 2019). However, when a plate was placed on the antero-lateral side of the cricket, the direction in which the cricket moved was shifted to the opposite side of the plate for stimuli that were applied from the rear (Fig. 2), and the movement was shifted backwards for lateral stimuli, which were applied from the opposite side of the plate (Fig. 3). These shifts in the walking direction are thought to indicate avoidance of collisions with objects perceived as walls. However, the treadmill system we used was controlled in open-loop, so no object actually moved as the cricket walked. Therefore, it was still possible that the animal felt discomfort owing to mismatch between the feedback signals of self-motion and the sensory inputs. Nevertheless, the escape trajectory was clearly displaced in the direction of avoiding the object, suggesting that the crickets would alter their behavior in anticipation of ‘possible’ collision with obstacles.

Cockroaches walking near a wall maintain a constant distance while keeping their antennae in contact with the wall (Camhi and Johnson, 1999), and surgically shortening the cockroach's antennae increases the collision rate with the wall (Baba et al., 2010). The results from the present study, however, showed no difference in the spontaneous walking before the air-puff stimulation with and without the wall at the near position (Fig. S3). This meant that the crickets with their antennae in contact with the wall did not reflexively keep the constant distance from the wall, nor did they move towards the wall (Camhi and Johnson, 1999; Okada and Toh, 2000). In contrast, the crickets flexibly altered their escape trajectory to avoid collisions, depending on the angle of airflow (stimulus), even if the same obstacles are placed in an identical position. For example, when the plate was placed at the near position on the lateral side of the crickets, their forward movement triggered by the stimulus applied from behind was biased toward the opposite side of the plate, while the lateral movement induced by the stimulus from the opposite side of the plate was altered toward the back (Figs 4 and 5). This suggests that crickets could modulate their behavior depending on the spatial relationship between the stimulus directly triggering the behavior and the environment, that is, the spatial context. It has been reported that descending signals from the cricket brain are necessary to regulate the escape direction (Oe and Ogawa, 2013). Some descending neurons sensitive to artificially caused antennal movement have been identified in the cricket brain (Gebhardt and Honegger, 2001). The mechanosensory information presented by the insect's antennal system is possibly processed by the brain and used to control their movement for successful escape via descending neurons.

The changes in escape trajectory depended on the location and orientation of the objects. The crickets moved toward a free space in the absence of obstacles (Figs 4 and 5). This fact suggests the ability of crickets to perceive the arrangement of objects in the surrounding space. Interestingly, the escape response to the airflow from the rear was delayed and the forward movement was suppressed when the plate was positioned at the center in front of the cricket (Fig. 5E). Because running forward possibly caused a collision with the wall in the front, the crickets might have delayed their decision to start their escape movement. In contrast, the plate positioned to one side in front of the animal biased the escape toward the wall-free side but did not affect the reaction time. This implies that the response delay and the reduction in the walking distance are not simply due to the reactive inhibitory effects of the mechanosensory inputs from the antennae. It has been reported that high-frequency sounds that hint at the presence of predators reduce the response probability of wind-elicited escape (Fukutomi and Ogawa, 2017). Our results imply that crickets can flexibly change their escape behavior depending not only on acoustics but also on the spatial contexts sensed by the antennal system in the surrounding space. The crickets integrate the sensory inputs of multiple modalities to perceive the surrounding context and make decisions for an appropriate and successful escape.

### Crickets locate objects by using edge detection with one antenna

Comparing the inputs from the left and right antennae is useful for obtaining a more accurate picture of the surrounding environment. For example, in navigation to localize an odor source, insects sample chemicals using a pair of antennae as chemical sensors and compared the sensory inputs to orient (Eiras and Jepson, 1994; Willis, 2008; Takasaki et al., 2012). The bilateral comparison of antennal inputs has also been reported for mechanosensory cues. Crayfish have been reported to compare tactile inputs from both antennae to determine the turning direction (McMahon et al., 2005). However, our results indicated that crickets did not necessarily need to compare tactile information between the left and right antennae to locate the front obstacles, because the plate placed to one side in front of the cricket altered the direction of movement and turn angle even though the one antenna contralateral to the plate was ablated (Fig. 6E). This meant that the inputs from one antenna contacted with the object provided information sufficient for object localization even if the other antenna was lost.

However, it remains possible that crickets use bilateral comparison of left and right antennal inputs to locate the object in front. In the unilaterally antenna-ablated crickets, the walking trajectory was slightly biased toward the intact side even under the no-wall condition, although their walking directions were not significantly different from those before the ablation (Fig. 6E, Table S1H). This may be because the ablated antenna provided no information about the presence or absence of an object, but the intact antenna actively provided ‘no obstacle’ information, so they were oriented toward the intact side under the no-wall condition. The perception of the absence of objects is one of the important functions of active sensing, for which animals repeatedly scan the environment by moving their sensory organ voluntarily (Bermejo et al., 2005; Nelson and MacIver, 2006). In addition, no significant difference in the walking direction between control of the intact cricket and wall-presented condition of the unilaterally ablated cricket suggests additional effects of the one-sided wall in intact crickets, which may result from bilateral comparison of left and right antennal inputs indicating the ‘presence’ and ‘absence’ of wall, respectively. It is likely that insects perceive the surrounding space, including object location, based on edge detection and the perception of absence using active sensing with their antennal mechanosensory system.

## Supplementary Material

Supplementary information
