# Supplementary material for: Spatial perception mediated by insect antennal mechanosensory system
Source: J Exp Biol. 2022 Feb 24;225(4):jeb243276. doi: 10.1242/jeb.243276 (PMC8920036; doi:10.1242/jeb.243276)
Supplement: Supplementary information [file jexbio-225-243276-s1.pdf]

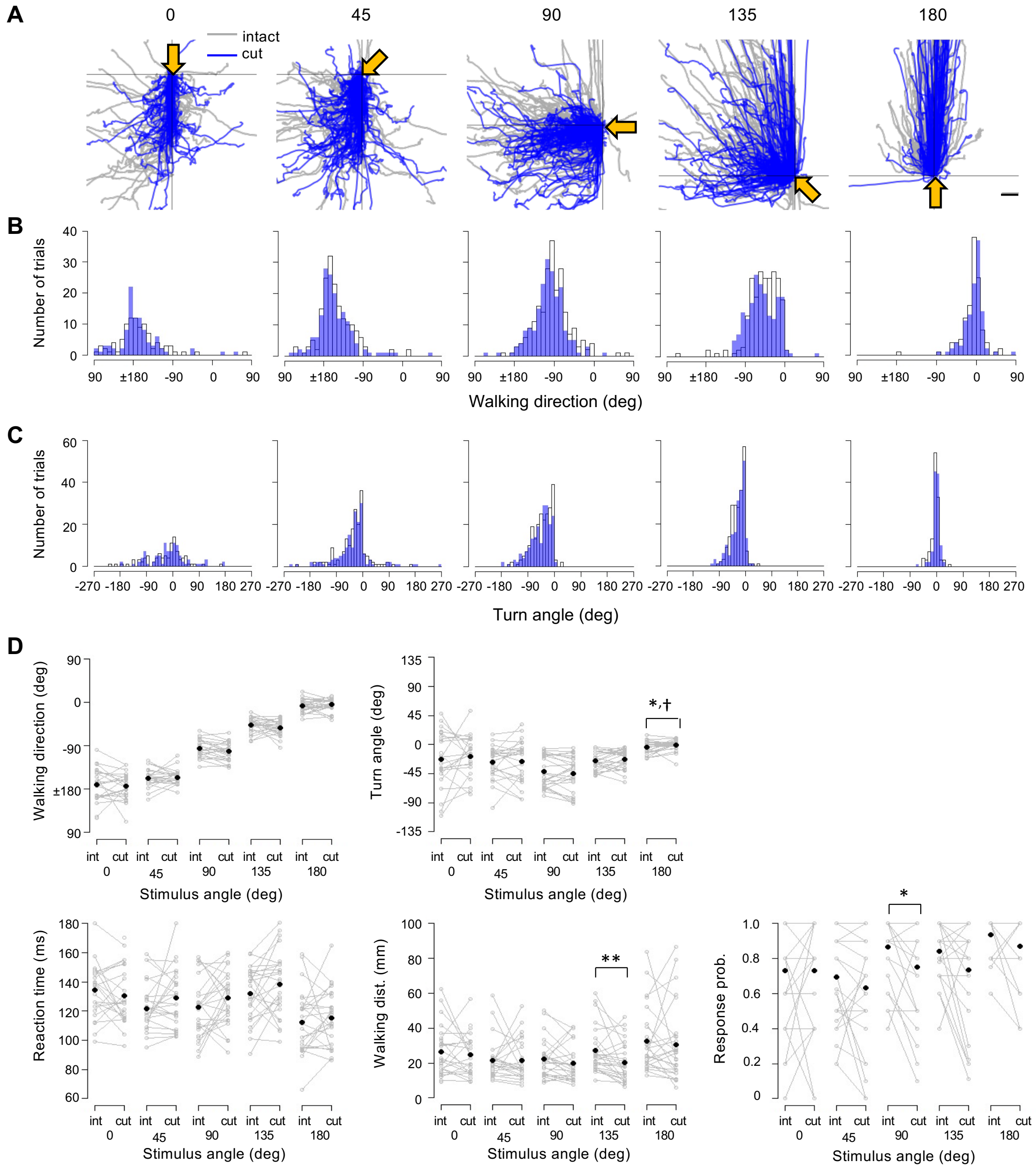

Fig. S1. Effects of bilateral antennae ablation on wind-elicited escape behavior.

A. Walking trajectories in the initial response to a puff of air from different angles. The data of the responses to the stimuli from the right and left sides were combined (45° and –45°, 90° and –90°, 135° and –135°). Gray traces show the trajectory of the intact

Journal of Experimental Biology • Supplementary information

crickets and the blue traces show the trajectory of the crickets whose antennae were resected bilaterally. Scale bar shown in the right (180°) panel indicates 10 mm. **B, C.** Distributions of walking direction (B) and turn angle (C) in the initial responses to stimuli from different angles. Open and blue bars indicate data from crickets with intact antennae and from antennae-ablated crickets, respectively. **E.** Differences between the different conditions in the five locomotion parameters including, walking direction, turn angle, reaction time, walking distance, and response probability. N = 26 individuals. Five trials were performed for each stimulus angle for each individual. Gray open circles connected by lines indicate mean values for all trials in each individual. Black dots denote average across the population for each condition. There was no significant difference in the walking direction between the various stimulus angles. The other parameters were also not significantly different for stimulus angles that were close to the antenna. * $P < 0.05$, ** $P < 0.01$. (Fisher's nonparametric test, and Wilcoxon signed-rank test). † $P < 0.05$ (Wallraff test).

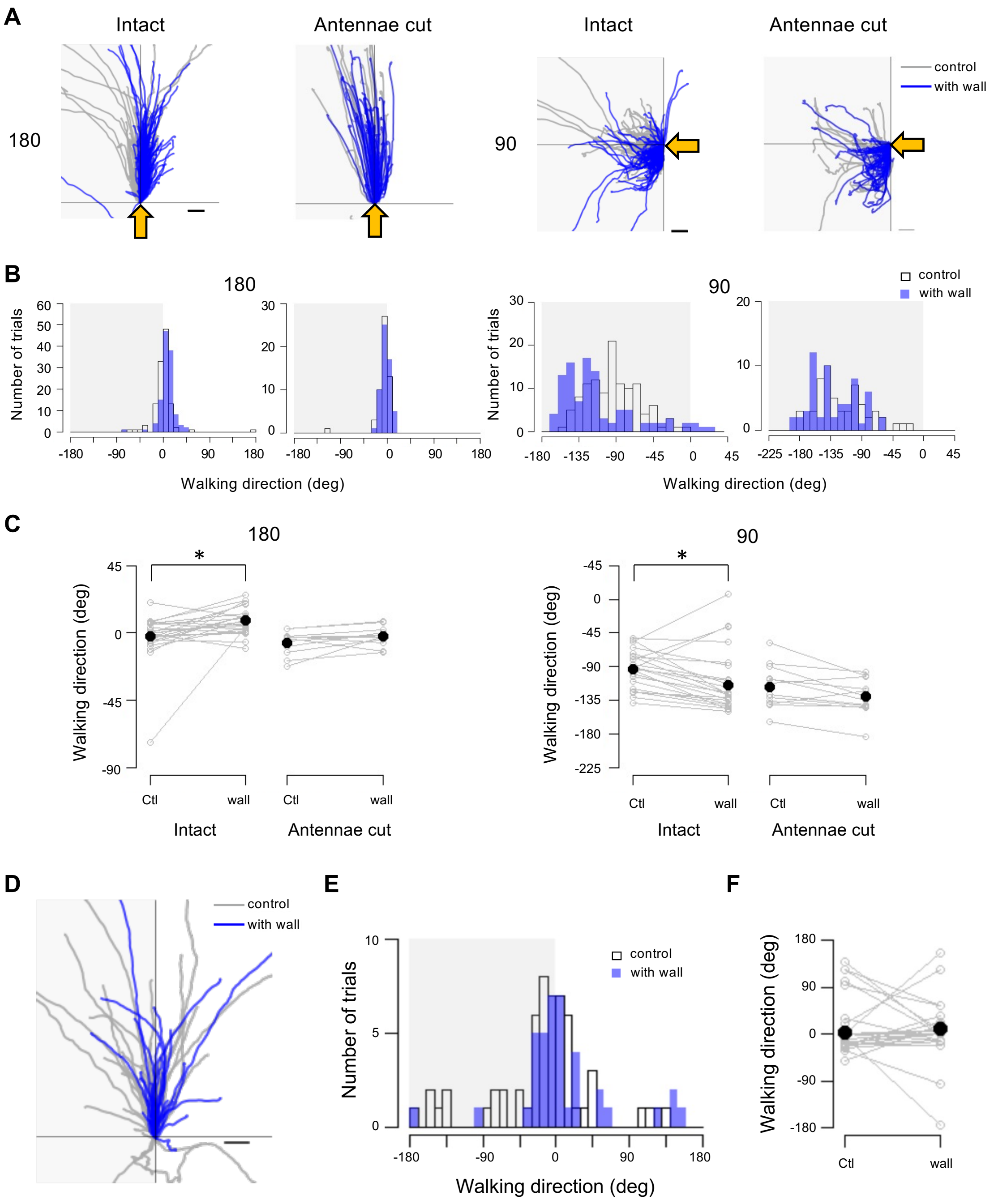

Fig. S2. No influence of the air turbulence on the escape behavior and no impacts of the antennal stimulation on the spontaneous walking.

A. Walking trajectories in the initial response to a puff of air from behind (180, left) or

Journal of Experimental Biology • Supplementary information

lateral side (90, right) of the intact crickets and those of which antennae were resected bilaterally. The data in intact crickets are the same as those shown in figures 2 and 3. Gray traces show the trajectories under the control condition without any objects. Blue traces show the trajectories when the plate was placed at the near position on the contralateral side to the airflow stimulus. Scale bars indicate 10 mm. **B.** Distributions of the walking direction in the initial responses to stimuli from behind (180, left) or side (90, right) of the cricket. Open and blue bars indicate data from the control and when the wall was present, respectively. **C.** Walking directions under different conditions in intact and bilaterally antennae-ablated crickets. N = 24 and 12 individuals for the intact and antennae-ablated crickets, respectively. Five trials were performed for each condition for each individual. Gray open circles connected by lines indicate mean values for all trials in each individual. Black dots denote average across the population for each condition. * $P < 0.05$ (Fisher's nonparametric test). When the antennae were ablated bilaterally, there was no significant difference in the walking direction between the presence and absence of the wall. **D.** Trajectories of each bout during the spontaneous walking before the airflow stimulation under control (no wall) and near-wall conditions. All traces were aligned with the position and head direction at the bout start. The criteria of the start and end points for the walking bout was the same as that for the initial response to the airflow stimulus. Gray and blue traces indicate the trajectories under the control and near-wall conditions, respectively. **E.** Distributions of the walking direction for spontaneous walking bout under different conditions. Open and blue bars indicate data from the control and when the wall was present,

Journal of Experimental Biology • Supplementary information

respectively. **F**. Walking directions under different conditions. Gray open circles connected by lines indicate t mean values for all walking bouts in each individual. Black dots denote average across the population for each condition. There was no difference in direction of the spontaneous-walking bouts (*P* = 0.1607, Fisher's nonparametric test). N = 24 individuals. The data in **D-F** were obtained from trials shown in figure 2.

Journal of Experimental Biology • Supplementary information

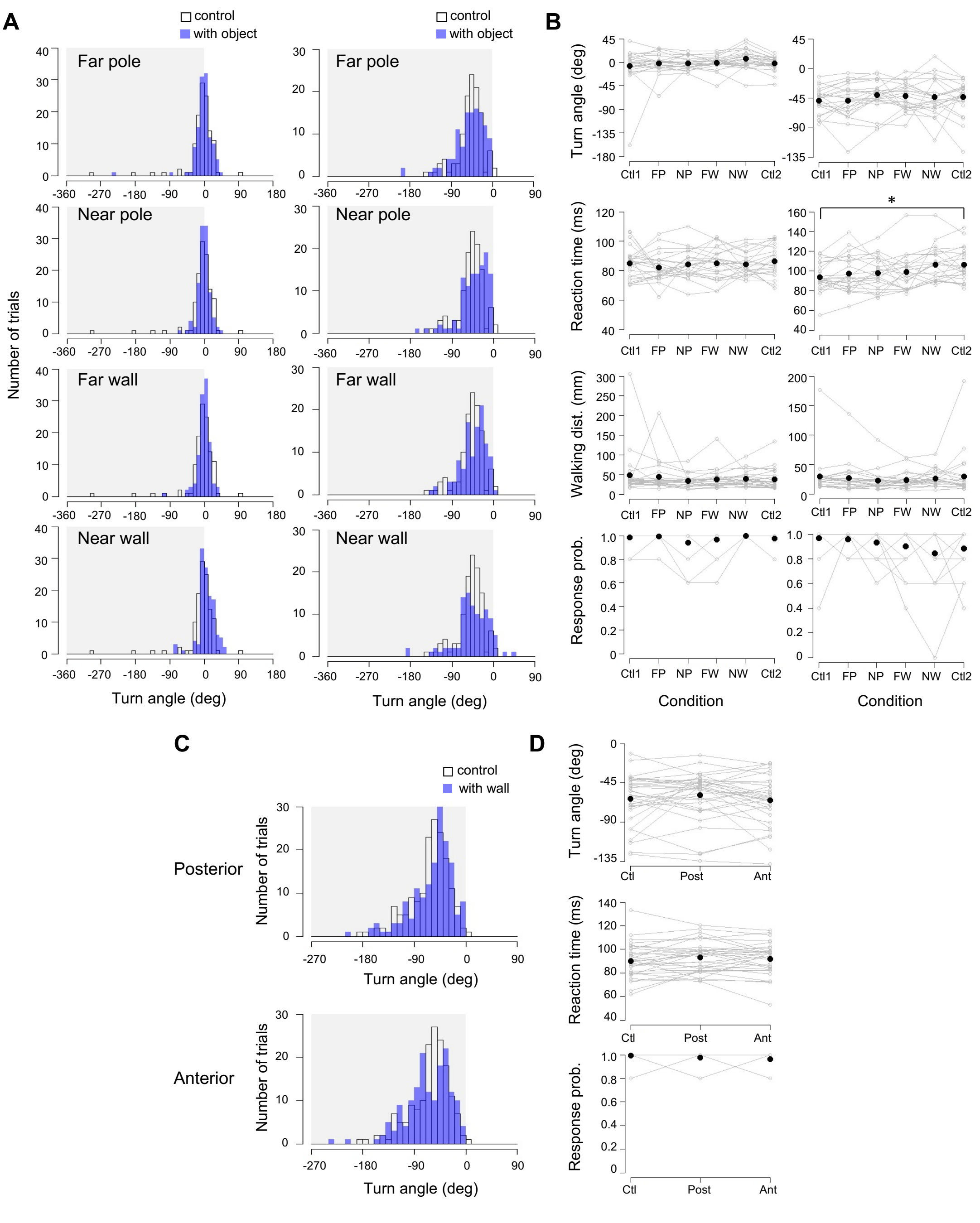

Fig. S3. The locomotion parameters that were not significantly affected by antennal mechanosensory inputs in the escape response to airflow.

A. Distributions of turn angle under different conditions shown in figures 2 and 3. Open and blue bars indicate the data from the control and antennal-stimulation conditions,

Journal of Experimental Biology • Supplementary information

respectively. **B.** Locomotion parameters including turn angle, reaction time, walking distance, and response probability under different conditions shown in figures 2 and 3. Left and right columns in A and B indicate the results for the stimulus from behind and the side contralateral to obstacles, respectively. N = 24 individuals. Data for the control condition in A and B were obtained for each individual twice, at the beginning (Ctl1) and at the end (Ctl2) of the experiment. FP, far pole; NP, near pole; FW, far wall; NW, near wall. **C**. Distributions of turn angle under different conditions shown in figure 4. Open and blue bars indicate the data from the control and antennal-stimulation conditions, respectively. **D.** Locomotion parameters including turn angle, reaction time, and response probability under different conditions shown in figure 4. N = 33 individuals. Ctl, control; Post, posterior; Ant, anterior. Five trials were implemented for each condition for each individual. Gray open circles connected by lines indicate mean values for all trials in each individual. Black dots denote average across the population for each condition.

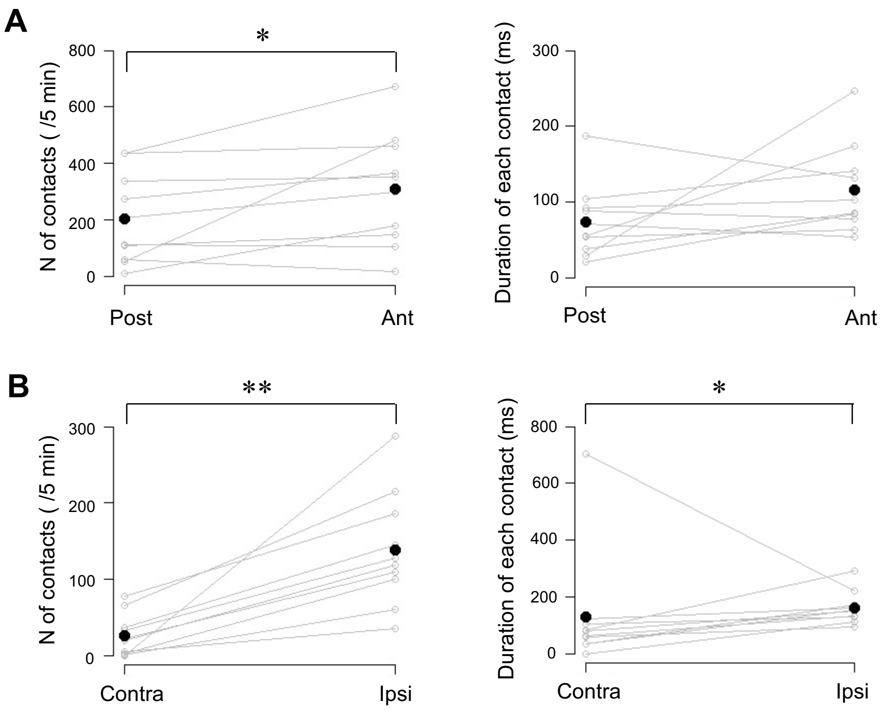

Fig. S4. Frequency and duration of contacts made by the antenna with the plate placed on the side or in front of cricket.

A. The total number of contacts (left) and duration of a single contact (right) made by the antenna with the plate positioned anteriorly or posteriorly on the side of the cricket. **B.** The total number of contacts (left) and duration of a single contact made by the ipsilateral or contralateral antenna to the plate positioned to one side in front of the cricket. N = 10 individuals for each measurement. The total sampling duration for each condition was 5 min. Gray open circles connected by lines indicate mean values for all trials in each individual. Black dots denote average across the population for each condition. * $P < 0.05$, ** $P < 0.01$ (Wilcoxon rank-sum test). Ctl, control; Post, posterior; Ant, anterior; Contra, contralateral antenna to the wall, Ipsi, ipsilateral antenna to the wall.

Journal of Experimental Biology • Supplementary information

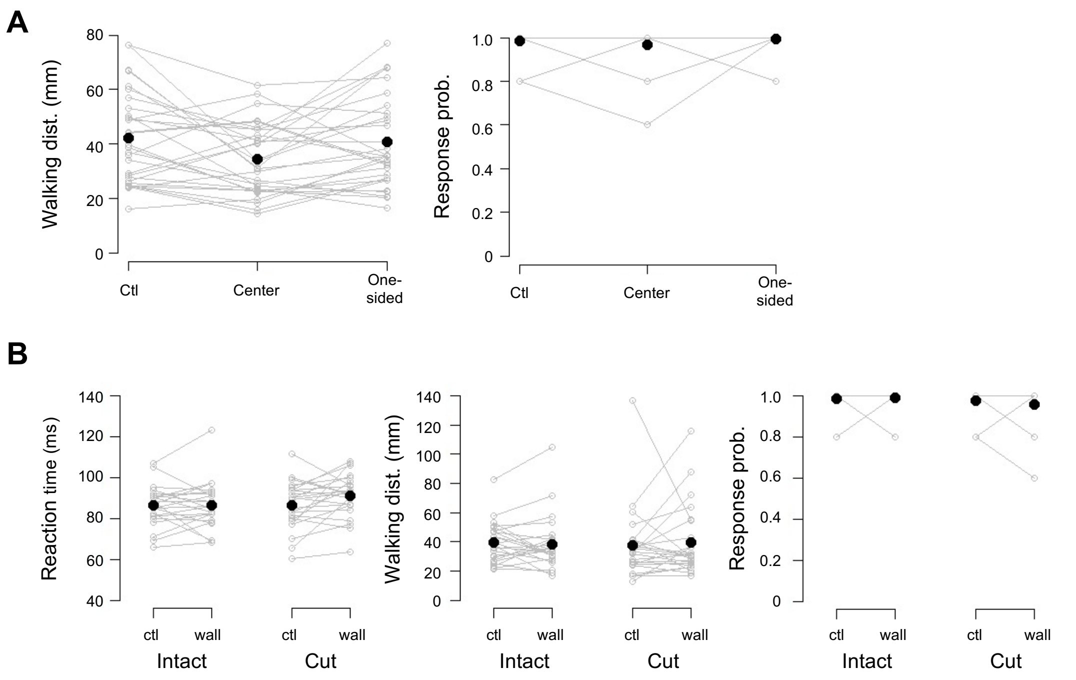

Fig. S5. Parameters that were not significantly affected by the front wall.

A. Walking distance and response probability when the wall was placed at medial and lateral positions in front of the intact cricket. N = 30 individuals. Related to Fig. 5. **B.** Reaction time, walking distance, and response probability in the intact and unilaterally antenna-ablated cricket. N = 24 individuals. Related to Fig. 6. Five trials were implemented for each condition for each individual. Gray open circles connected by lines indicate mean values for all trials in each individual. Black dots denote average across the population for each condition.

Table S1. Statistical results

A

Stimulus angle	Walking direction		Turn angle		Reaction time	Walking distance	Response probability
	Median	Angular dispersion	Median	Angular dispersion			
0º (front)	0.4767	0.4009	0.7287	0.1499	0.4936	0.789	0.752
45º	1	0.969	0.7362	0.0570	0.1787	0.5964	0.131
90º (side)	1	0.9126	0.2173	0.9126	0.1651	0.0890	0.0132
135º	0.5791	0.7143	0.4678	0.8981	0.1499	0.0032	0.0856
180º (behind)	0.2673	0.6212	0.0176	0.0422	0.3802	0.4227	0.1228

B

Tested conditions	Walking direction		Turn angle		Reaction time	Walking distance	Response probability
	Median	Angular dispersion	Median	Angular dispersion			
Ctl / Far pole	0.0833	0.7105	0.5457	0.5919	0.6231	0.8996	1
Ctl / Near pole	0.2482	0.5777	0.8553	0.4095	0.9441	0.0647	0.05447
Ctl / Far wall	0.0833	0.5637	0.4223	0.1671	0.8996	0.3449	0.1736
Ctl / Near wall	0.0195	0.7415	0.1243	0.665	0.7469	0.8334	1
Ctl 1 / Ctl 2	0.2482	0.7728	0.5088	0.5499	0.6033	0.1974	0.4237

C

Tested conditions	Walking direction		Turn angle		Reaction time	Walking distance	Response probability
	Median	Angular dispersion	Median	Angular dispersion			
Ctl / Far pole	0.2482	0.6062	0.8143	0.2399	0.6022	0.4308	0.5877
Ctl / Near pole	0.0833	0.6501	0.0814	0.9343	0.2818	0.1253	0.2542
Ctl / Far wall	0.2482	0.7728	0.2727	0.2655	0.4803	0.2143	0.08898
Ctl / Near wall	0.0281	0.4187	0.6655	0.5513	0.0832	0.8085	0.1019
Ctl 1 / Ctl 2	0.5637	0.5919	0.1202	0.7887	0.0316	0.1153	0.2208

D

Tested conditions	Stimulus angle	Antennae condition	Walking direction	Number of individuals
Ctl / Near wall	180º (behind)	intact	0.0195	24
		ablated	0.1025	12
	90º (side)	intact	0.0281	24
		ablated	0.4142	12

Journal of Experimental Biology • Supplementary information

E

Tested conditions	Walking direction		Turn angle		Reaction time	Walking distance	Response probability
	Median	Angular dispersion	Median	Angular dispersion			
Ctl / Posterior	0.0802	0.1047	0.6088	0.6306	0.1391	0.9578	0.233
Ctl / Anterior	0.0041	0.0917	0.672	0.6768	0.1085	0.0017	0.0726
Post / Ant	0.2184	0.6862	0.2418	0.9949	0.396	< 0.0001	0.5653

F

Tested conditions	Frequency	Duration
Posterior / Anterior (Wall position)	0.0273	0.1934
Ipsi / Contra (Side of antenna)	0.0002	0.0058

G

Tested conditions	Walking direction		Turn angle		Reaction time	Walking distance	Response probability	Forward distance
	Median	Angular dispersion	Median	Angular dispersion				
Ctl / Center	0.3017	0.0001	0.3707	0.0066	0.0279	0.0524	0.2031	0.004
Ctl / One-sided	< 0.0001	0.1379	< 0.0001	0.1379	0.7324	0.4771	0.7728	0.1642
Center / One-sided	0.0058	0.0266	0.0001	0.0266	0.9816	0.0832	0.2031	0.0524

H

Tested conditions	Antennae condition	Walking direction		Turn angle		Reaction time	Walking distance	Response probability
		Median	Angular dispersion	Median	Angular dispersion			
Ctl / Wall	Intact / Intact	< 0.0001	0.2599	< 0.0001	0.4705	0.7257	0.7683	0.7728
	Intact / Ablated	0.1255	0.0284	0.1268	0.6062	0.1492	0.7257	0.3447
	Ablated / Intact	< 0.0001	0.2745	0.0002	0.6501	0.7683	0.2643	0.4237
	Ablated / Ablated	0.0234	0.0909	0.0051	0.7887	0.0564	0.5457	0.3741
Ctl /Ctl	Intact / Ablated	0.0837	0.2655	0.1515	0.6650	0.6033	0.0951	0.7656
Wall / Wall	Intact / Ablated	0.1255	0.5362	0.2369	0.6501	0.1268	0.8115	0.2031

Tested conditions	Angular difference in walking direction	Angular difference in turn angle
Intact / Ablated	0.0526	0.2634

A. Statistical results to test effects of bilateral antennae ablation on locomotor parameters in the escape response to air-puff stimulus from 8 different angles. $N = 26$. The data were represented in Fig. S1. **B**. Statistical results to test effects of object shape and location on locomotor parameters in the escape response to air-puff stimulus from behind. $N = 24$. The data were represented in Fig. 2 and Fig. S3. **C**. Statistical results to test effects of object shape and location on locomotor parameters in the escape response to air-puff stimulus from the side contralateral to the objects. $N = 24$. The data were represented in Fig. 3 and Fig. S4. **C**. Statistical results to test air-turbulence effects of walls placed at near position on walking direction in the escape responses. The data were represented in Fig. S2. **D**. Statistical results to test positional effects of side walls on locomotor parameters in the escape response to air-puff stimulus from the side contralateral to the objects. $N = 24$. The data were represented in Fig. 4 and Fig. S5. **E**. Statistical results to test positional effects of side walls on locomotor parameters in the escape response to air-puff stimulus from the side contralateral to the objects. $N = 24$. The data were represented in Fig. 4 and Fig. S5. **F**. Statistical results to test differences in contacts made by the antenna with the walls placed at different positions. $N = 10$. The data were represented in Fig. S6A. **G**. Statistical results to test positional effects of walls placed in front of crickets on locomotor parameters in the escape response to air-puff stimulus from behind. $N = 30$. The data were represented in Fig. 5 and Fig. S7A. **H**. Statistical results to test effects of the walls placed toward one side in front of unilaterally antenna-ablated crickets on locomotor parameters in the escape response to air-puff stimulus from behind. The data were represented in Fig. 6 and Fig.

S7B. The numbers indicate *p*-values provided by Fisher's nonparametric test for the median of walking direction, Wallraff test for the angular dispersion of walking direction and turn angle, and Wilcoxon signed-rank test for other parameters including differences in the frequency and duration of the antennal contacts (F) and angular differences in walking direction and in turn angle (H) with Bonferroni correction.

Journal of Experimental Biology • Supplementary information
